# Student experiences and perspectives on decolonising global health education: a qualitative study across five Nordic countries

**DOI:** 10.1080/16549716.2025.2512624

**Published:** 2025-06-12

**Authors:** Jennifer J. Infanti, Jessica Omija Korpela, Chloe Maria Stephenson, Astrid Blystad, Jane Brandt Sørensen, Jónína Einarsdóttir, Mekdes Kebede Gebremariam, Geir Gunnlaugsson, Tobias Herder, Meri Koivusalo, Jesper Löve, Karima Lundin, Mikko Perkiö, Soorej Jose Puthoopparambil, Klas-Göran Sahlen, Marte Emilie Sandvik Haaland, Jon Petter Stoor, Salla Atkins

**Affiliations:** a Department of Public Health and Nursing, NTNU–Norwegian University of Science and Technology, Trondheim, Norway; b Faculty of Social Sciences, Tampere University, Tampere, Finland; c Centre for International Health, Department of Global Public Health and Primary Care, University of Bergen, Bergen, Norway; d Department of Public Health, University of Copenhagen, Copenhagen, Denmark; e Faculty of Sociology, Anthropology and Folkloristics, School of Social Sciences, University of Iceland, Reykjavík, Iceland; f Department of Community Medicine and Global Health, University of Oslo, Oslo, Norway; g Sustainable Health Unit, Faculty of Medicine, University of Oslo, Oslo, Norway; h Social Medicine and Global Health, Department of Clinical Sciences, Lund University, Malmö Sweden; i School of Public Health and Community Medicine, University of Gothenburg, Gothenburg, Sweden; j Department of Global Public Health, Karolinska Institutet, Stockholm, Sweden; k Global Health and Migration Unit, Department of Women’s and Children’s Health, Uppsala University, Uppsala, Sweden; l Department of Epidemiology and Global Health, Umeå University, Umeå, Sweden

**Keywords:** Curricula reform, pedagogical reform, power dynamics, student perspectives, global health equity

## Abstract

**Background:**

Decolonisation has become a global health priority, addressing inequities rooted in colonial histories that continue to shape power dynamics and knowledge systems. Nordic global health programmes bring together students and faculty from diverse backgrounds in a region defined by inclusive ideals but shaped by underexamined colonial legacies. This context offers a valuable setting to examine how decolonial perspectives are integrated or overlooked in global health education.

**Objective:**

To explore students’ perspectives on decolonisation in global health education, focusing on their understanding, experiences, and views on potential pedagogical change.

**Methods:**

A qualitative study involving 72 students from Nordic countries and other world regions, enrolled in global health programmes at 11 academic institutions across five Nordic countries. Fourteen focus group discussions were conducted, and the data were analysed using qualitative content analysis principles.

**Results:**

Students shared nuanced understandings of systemic power imbalances in global health practice and education and expressed the need for structural changes. They identified gaps in curricula and pedagogy, including limited integration of decolonial perspectives and inequities in knowledge production. Students called for more inclusive and culturally relevant curricula that reflect diverse contexts. They emphasised student agency in shaping education while acknowledging barriers such as institutional biases and inconsistent faculty engagement.

**Conclusions:**

Decolonising Nordic global health education is a long-term process requiring sustained institutional commitment. Student-informed strategies include embedding reflexivity into curricula, engaging with Nordic colonial histories, and designing reciprocal international learning arrangements. While context-specific, these findings may inform broader efforts to decolonise global health educational practices.

## Paper Context

**Main findings:**

Nordic global health students perceive colonial legacies to persist in curricula, pedagogy, and institutional practices, undermining the equitable representation of diverse knowledge in their study programmes

**Added knowledge:**

This study reveals students’ experiences with and perspectives on decolonisation in Nordic global health education and offers concrete strategies to embed diverse knowledge systems and foster equity.

**Global health impact for policy and action:**

These insights call for structural reforms in global health education, including curriculum diversification, reciprocal partnerships, and enhanced faculty training to promote inclusive and decolonised global health practices and learning environments.

## Background

Global health extends beyond national borders, addressing transnational health challenges and broad social, political, and environmental determinants of health [1,2]. Evolving from its colonial and tropical medicine roots to international and now global health, the field has increasingly recognised how historical power dynamics shape present-day global health inequities [3–5]. This recognition has brought decolonisation to the forefront of global health educational and practice agendas today [6,7].

University-based global health education and research has predominantly developed in high-income countries (HICs), where such programmes have rapidly expanded and become popular among both national and international students [8]. Despite significant access barriers – such as high tuition fees, stringent visa requirements, and the need for proof of substantial financial resources to qualify for enrolment – these programmes continue to attract students from low- and middle-income countries (LMICs).

The curricula in many of these programmes often focus on health challenges in LMICs, while overlooking local innovations or structural inequalities within HICs themselves [9–13]. Decolonial challenges in global health education thus include addressing the dominance of HIC-based perspectives, which frequently marginalise LMIC expertise and knowledge systems, reinforcing inequities in representation, agency, and leadership [14–17]. These dynamics are further compounded by the continued concentration of funding, influence, and institutional authority in HIC settings, shaping who produces knowledge and whose voices are heard in global health discourse [17].

In parallel, widely used geopolitical framings – for example, ‘global north’ and ‘global south’ or ‘HIC’ and ‘LMIC’ – although useful in analysing global inequalities, also risk oversimplifying complex realities [12,15]. Critics argue that these terms reflect Western constructions of value, privileging economic metrics over other dimensions of wealth, such as cultural knowledge, creative capacity, and community strength [17–19]. Responding to these critiques, decolonising global health education calls for inclusive, reflexive approaches that expand access, elevate diverse epistemologies, question dominant classifications, and foster equitable, reciprocal partnerships between institutions globally.

While there is increasing global discourse around decolonisation, Nordic global health education programmes have only recently begun to engage in these debates. This may reflect a broader lack of reckoning with coloniality in Nordic academic and institutional contexts [20]. Nordic institutions, situated in contexts associated with egalitarian welfare traditions and low regional income inequality, tend to underemphasise how their historical roles and contemporary advantages are embedded in broader colonial systems – a pattern also observed in other settings. Nordic countries have benefited structurally and economically from global systems of colonisation – including participation in transatlantic slavery, colonial trade networks, and the marginalisation of Indigenous Sámi and Inuit populations – legacies that are often absent from their global health curricula. This absence may be due not only to limited historical reckoning, but also to gaps in faculty awareness and training, curricular space, or institutional prioritisation.

Global health programmes in the Nordic region are typically structured around interdisciplinary training in topics such as maternal, child, and adolescent health; mental health; health systems; migration; humanitarian emergencies; climate change; epidemiology and other research methods; social determinants of health; and health equity. Many emphasise student-centred learning, reflecting broader educational values in the region. Faculty tends to be trained in clinical medicine, public health, or social sciences, with varying levels of exposure to critical pedagogy or postcolonial theory. The programmes are delivered in English to small cohorts (usually 15–40 students) that include a mix of Nordic and international students from both HICs and LMICs. While public resources fund free tuition for Nordic and European Union (EU) citizens, participation of students from other regions is restricted by exchange programmes, national tuition policies, and immigration systems. As such, the Nordic education context – defined by inclusive ideals, but shaped by structural inequities – provides a compelling setting for examining how decolonial perspectives are integrated (or overlooked) in global health training.

In recent years, student awareness and activism have increased, with growing calls to address Eurocentrism and colonial legacies in global health education. In some Nordic institutions, student associations and course-level initiatives have championed the inclusion of decolonial topics or demanded curricular reforms (for example, see [21]). However, the pace and depth of these efforts vary considerably, and little is known about how students themselves understand and navigate the broader discourse on decolonisation within their study programmes. This study thus explores students’ perspectives on decolonisation within global health education in 11 universities in five Nordic countries, focusing on their understanding, experiences, and views on potential pedagogical change. We offer a context-specific exploration of how decolonial aspirations are interpreted and engaged with by students within a particular regional and institutional setting. By doing so, we seek to identify actionable strategies for advancing more inclusive, equitable, and contextually relevant approaches to global health education, both within and beyond the Nordic region.

## Methods

### Study design and setting

This exploratory qualitative study focuses on student experiences and perspectives. It captures how students interpret and engage with decolonisation within the broader educational and institutional environments in which they are enrolled.

The study was conducted within the Nordic Network of Global Health Education, a collaboration initiated in 2015 to foster relationships among universities in the region offering global health education programmes. Study participants were students from 11 universities in five Nordic countries: Tampere University (Finland); Karolinska Institutet, University of Gothenburg, Lund University, Umeå University, and Uppsala University (Sweden); the Norwegian University of Science and Technology (NTNU), University of Oslo, and University of Bergen (Norway); the University of Copenhagen (Denmark); and the University of Iceland (Iceland).

### Study participants

We used purposive sampling, applying a maximum variation approach [22] to recruit students from diverse backgrounds and stages of study. Focus group invitations were shared via email and learning platforms at participating universities. Interested students then received an information sheet outlining the study, including a flyer with basic details on decolonisation in global health. All participants provided informed consent before engaging in focus group discussions (FGDs).

The final sample consisted of 72 students, enrolled primarily in bachelor’s- and master’s-level global health programmes or elective courses across the participating universities. Participants varied in age, gender, academic and regional background, and professional experience. Table 1 provides an overview of participant characteristics and sample diversity. These data are included for contextual purposes and were not used for subgroup analysis. Data were available for most participants, although some information was not registered due to institutional data-sharing restrictions or partially filled background information questionnaires.

**Table 1.** Demographic and educational characteristics of participants in eleven focus group discussions on decolonisation in global health.*

Number of participants	72	
**Age (years)**		
Mean (range)	31.5 (23–56)	
**Gender, n (%)**		
Male	17 (24)	
Female	41 (57)	
Not available	14 (19)	
**Programme of study, n (%)**		
Bachelor’s degree (BSc)	11 (15)	
Master’s degree (MSc)	34 (47)	
Doctoral degree (PhD)	3 (4)	
Other (e.g. medical school)	2 (3)	
Not available	22 (31)	
**Year in study programme, n (%)**		
First	18 (25)	
Second	17 (24)	
Third	5 (7)	
Fourth	1 (1)	
Graduated	9 (13)	
Not available	22 (31)	
**Region, n (%)**		
East Asia and Pacific	2 (3)	
Europe and Central Asia	21 (29)	
Latin America and Caribbean	4 (6)	
Middle East and North Africa	4 (6)	
North America	5 (7)	
South Asia	4 (6)	
Sub-Saharan Africa	13 (18)	
Not available	19 (26)	
**Prior work experience in global/public health, n (%)**		*Reported sources*
Yes	34 (47)	Research institutions; local/national non-governmental organisations (NGOs); international NGOs; civil society organisations; hospitals/healthcare facilities; government departments; public agencies; MedTech companies; news media organisations; other relevant settings
No	21 (29)	
Not available	17 (24)	
**Prior awareness of decolonisation, n (%)**		*Reported sources*
Yes	48 (64)	Internet/social media; family/friends/school; radio/TV; books/newspapers/magazines; course literature; other academic literature; seminar/course activity; organisation debates; student associations; symposiums; outreach/flyers; webinars; international student festivals/events; podcasts; and other sources/resources
No	8 (11)	
Not available	18 (25)	

*Not available’ values reflect privacy regulations, incomplete responses, or participant non-response to the background questionnaire.

### Data collection

FGDs were used to efficiently engage a large and diverse sample of students from each university, while also allowing participants to reflect on and respond to one another’s perspectives. This method supported the emergence of shared, contested, and evolving views, offering insights into both individual and collective knowledge and experiences relevant to the study aims [23]. The FGDs were guided by open-ended questions and prompts exploring students’ motivations for studying global health, their understanding of decolonisation, and their experiences with how it is addressed in global health teaching. Additional topics included the relationships between decolonisation and key concepts in global health such as equity, anti-racism, and intersectionality; students’ anticipated roles and values as future global health practitioners; and potential recommendations for integrating decolonial perspectives into teaching, research, and practice. The FGD guide was developed collaboratively by the research team and used consistently across all sites. It was informally pre-tested by means of being presented at a doctoral seminar for students in global health and related social science fields at Tampere University. Their feedback was helpful in improving the relevance, clarity and flow of the questions. The session was not recorded or included in the study data.

We conducted 14 digital FGDs via Zoom (Zoom Video Communications, Inc., Version 5.15.0, 2023) between June and September 2023. Eight universities held one FGD each, while three universities each conducted two. Each session involved five to eight students, except for one session that had only a single participant.

The majority of FGDs (11 out of 14) were facilitated by a PhD candidate unaffiliated with the students, herself a former international master’s student in global health and trained in qualitative methods. This helped minimise potential power dynamics between the facilitator and participants and encouraged open discussion. The remaining three FGDs were moderated locally by a faculty member. At each university, a local co-moderator – typically, a faculty member – was also present to provide logistical support and demonstrate institutional engagement without actively shaping the discussion.

We recognised that group dynamics might also be influenced by factors such as nationality, region of origin, and gender. In most cases, students were already familiar with one another, having studied together for one year or more. To promote inclusive participation, the lead facilitator used standard focus group techniques, such as actively inviting contributions from quieter participants and encouraging a range of perspectives. Co-moderators helped create a comfortable and respectful discussion environment, while maintaining a low profile. Each session lasted 60–90 minutes and was audio recorded. Transcripts, supplemented by notes taken during and immediately after each session, were used for analysis.

### Data analysis

The analysis followed principles of qualitative content analysis (QCA) [24,25]. To manage the extensive dataset and ensure consistency across the co-author team, we implemented a structured, collaborative process. Initially, two co-authors (JOK, CMS) independently coded the same two FGD transcripts. They began by reading each transcript thoroughly, identifying and dividing the text into meaning units relevant to the research question, condensing these units while retaining their core meaning, and labelling them with codes. This initial coding framework was validated and refined in consultation with the last author (SA), who supervised the work and ensured adherence to established QCA principles throughout. This coding framework was then applied to the remaining transcripts.

As the analysis progressed, the same two co-authors (JOK, CMS) grouped the codes into preliminary sub-categories and manifest categories based on their similarities and differences relative to other codes. The manifest categories were then organised into preliminary themes, which were further refined in consultation with the last author (SA).

Preliminary results were discussed during a two-day workshop with the larger co-author group, and feedback from this session guided further refinements. A subset of four different co-authors (AB, KL, SJP, MP) then restructured the manifest categories to improve analytical clarity and ensure alignment with the coding framework. Building on the manifest categories, they also identified latent themes [26]. This group drafted the analytical results, reviewing and refining them through several rounds of feedback from all co-authors to ensure clarity and coherence. This process ensured that QCA principles – such as systematic coding, category abstraction, and interpretation of latent content – were applied consistently across the full dataset. Table 2 illustrates the analytical process, showing the progression from a meaning unit in a transcript to a theme.

**Table 2.** Example of the analytical process, illustrating the progression from a meaning unit to a theme.

Meaning unit	Condensed meaning unit	Code	Category	Theme
“How you understand decolonisation is uh … has to do with regimes of knowledge and relationships, like changing power relationships in armies between north and south, between Black and White, between Latinos and … and White people”.	Decolonisation involves knowledge regimes and shifting power relationships between north and south, as well as racialised power hierarchies.	Understanding decolonisation can be linked to understandings of power relations	Connecting decolonisation to persistent power imbalances	Challenging power imbalances to decolonise global health

The analysis ultimately resulted in three main themes. These reflect students’ understanding of decolonisation; their critiques of how decolonial perspectives are (or are not) reflected in their global health education; and their understanding of their own role in advancing decolonisation within Nordic global health programmes. The following section presents the themes, supported by illustrative quotes to provide depth and context. The presentation is framed around students’ collective perspectives, experiences, and insights, rather than individual differences based on university or study programme, gender, nationality or other personal demographics.

## Results

The study’s findings are summarised in Table 3, grouped into three themes. The first theme, ‘Challenging power imbalances to decolonise global health’, captures students’ understanding of decolonisation as a way of addressing persisting global power imbalances rooted in colonial histories, spanning issues of knowledge ownership, research agenda-setting, funding allocation, and institutional and faculty attitudes. The second theme, ‘Aligning global health education with decolonisation’, highlights students’ critiques of their Nordic global health programme curricula and pedagogy, which they often see as misaligned with decolonisation aims. The third theme, ‘Advancing decolonisation through student-led initiatives’, focuses on students’ perceptions of their own role in fostering decolonisation within Nordic global health education.

**Table 3.** Identified themes and their corresponding categories in student perspectives on decolonisation in global health education.

THEME 1: Challenging power imbalances to decolonise global health	THEME 2: Aligning global health education with decolonisation	THEME 3: Advancing decolonisation through student-led initiatives
Questioning ownership and agenda-setting Connecting to persistent power imbalances Navigating contested mindsets Confronting racism and knowledge inequities	Integrating decolonial perspectives and challenging Eurocentrism in curricula Reflecting on educators’ attitudes and pedagogy Engaging with Nordic colonial histories and diversifying global health knowledge	Reimagining exchange programmes as reciprocal partnerships Driving change through student agency and activism

### Challenging power imbalances to decolonise global health

#### Questioning ownership and agenda-setting

Although we introduced students to the concept and foundational principles of decolonisation through the study’s information brochure, the FGDs revealed initial confusion about its meaning. Many discussions began with students seeking clearer definitions, and questioning who defines decolonisation, who leads related initiatives, and whether the discourse adequately represents historically marginalised groups. Some students expressed concerns that decolonisation risks becoming a ‘buzzword’, widely discussed but lacking practical impact. For example, one participant noted that many of these discussions take place in high-income countries, often without meaningful representation from formerly colonised nations. This led to scepticism about whether such conversations reinforce existing power structures rather than dismantling them.

#### Connecting to persistent power imbalances

As the conversations progressed, however, students showed a strong capacity to critically engage with decolonisation, linking it to systemic power imbalances. They observed how neo-colonial structures continue to shape global health leadership, policies, and systems, reinforcing inequities despite efforts to ‘rebrand’ the field. Students argued that decolonisation must involve a practical restructuring of global power relations:

What I think about the most in terms of decolonisation of global health is restructuring the existing power structures and relations between different countries and regions. I think that [although] global health is renamed [from its predecessor disciplines], it still retains many of the structures of colonial health. (Group 4)

Students viewed equity as fundamental to achieving decolonisation, linking it to the dismantling of systemic hierarchies and fostering inclusive participation:

Decolonising … [means] there is no upper power; we are all the same … let everyone be able to contribute … irrespective of their country of origin or their economic power or their political influence … So, when we’re decolonising the structures, it means we’re trying to achieve equity and equality. (Group 6)

For some, achieving equity also meant creating opportunities for historically marginalised communities to reclaim autonomy and integrating local knowledge systems:

Decolonisation … [means giving] people from the global south and ethnic minorities the opportunity to sit at the table … when decisions are being made … to actually … voice their opinions and be respected and listened to. (Group 11)

#### Navigating contested mindsets

While students agreed on the need to address systemic inequities, they debated the mindsets and intentions required for meaningful decolonisation. One concern raised was that some might use the decolonisation discourse to justify withdrawing resources:

You would be surprised how many global health practitioners in the global south are against the decolonising discourse. Many of them are against it, and for one reason: it’s because if we talk about this, then we [will] have less funding. (Group 8)

Others reflected on internalised beliefs, describing ‘colonised mindsets’ or ‘victim mentalities’, which they argued perpetuate dependency on powerful actors in the global north and undermine the capacity to advocate for locally driven priorities. Some students described how such beliefs are instilled from an early age and reinforced by broader societal narratives:

Growing up, you’ve been led to believe … that the other race is superior. So, whatever they say, that is what is true about yourself. (Group 7)

Communities tend to perceive themselves as victims, perpetuating this ‘third-world ideology’, that people from the west or people from developed countries have to help us. So, this is why the ‘white saviour’ perception still goes on. (Group 2)

At the same time, students cautioned against adopting reactionary, anti-north stances that might undermine constructive dialogue, acknowledging the risk of falling into polarised rhetoric.

#### Confronting racism and knowledge inequities

Students discussed how superiority narratives rooted in racist or otherwise discriminatory assumptions influence global health practices. They argued that attitudes of ‘knowing better’ reinforce colonial power structures and act as a central barrier to achieving equality, as envisioned by decolonisation. A key concern was the control over knowledge production. While students acknowledged that local teams are often included in contemporary global health research, they argued that ownership of research agendas and data frequently remains concentrated in the global north:

OK, we included ‘the locals’, and they were a part of constructing the project and everything. But when you find the information [research results], who has the power to interpret it, and in what way? (Group 4)

Beyond overt hierarchies, students also described how subtler forms of discrimination, such as cultural insensitivity, exclusion from decision-making, and derogatory stereotypes, continue to reinforce power imbalances. Consequently, many advocated for greater autonomy for local scholars to set research priorities, lead knowledge production, and control funding resources, ensuring that knowledge systems are shaped by those directly affected by global health challenges.

These discussions about persistent practical, material and discursive power imbalances in global health naturally led students to more specific conversations about how Nordic global health education programmes are either addressing or perpetuating these dynamics.

### Aligning global health education with decolonisation

We asked students to reflect on how Nordic global health education addresses decolonisation debates. In response, they highlighted insubstantial coverage of decolonisation topics in their programmes, the prevailing Eurocentric orientations of curriculum content and pedagogy, and a general lack of historical awareness. Further, they identified challenges and opportunities for integrating decolonial perspectives more effectively.

#### Integrating decolonial perspectives and challenging Eurocentrism in curricula

A pervasive sentiment among students was that decolonial topics were either superficially addressed or entirely absent from their curricula. When included, students mentioned the frequent confinement of decolonisation to isolated lectures, preventing deeper engagement across courses. This fragmented approach gave the impression that its importance was not prioritised:

All I remember is that we had a very brief conversation about it during the first course we had … and that was it, you know, then the discussion ended. We went on to study other issues. (Group 7)

Students called for a more structured and integrated approach, embedding decolonial perspectives across their programmes to create ongoing, meaningful dialogue:

[Teachers should] not just mention decolonisation in ‘the decolonisation lecture’, but also in the other modules, like in the nutrition module, in the sexual health module, like how [is] decolonisation related to every topic in the global health area. (Group 8)

Furthermore, students critiqued the dominance of Eurocentric (Western/American) perspectives in teaching materials, highlighting the underrepresentation of scholars and case studies from the global south. They called for a greater diversity of voices and content to better reflect global realities:

There’s like a few papers we have looked at in the class … that have been written by a non-white man, but it’s still so minuscule compared to everything else that we always read. (Group 2)

Although students acknowledged some efforts by educators to diversify curricula, they often felt that these were performative, tokenistic, or superficial, undermining their credibility and impact. Some also expressed frustration that decolonisation was sometimes dismissed as activism rather than a serious academic discussion. To address these concerns, they suggested training and awareness-raising for educators to be able to facilitate more informed and critical decolonial discussions:

We proposed … a kind of ‘train the trainers’ … to have in people who are experts in the field of decolonisation … so [our teachers] could also be trained on the topic. (Group 11)

#### Reflecting on educators’ attitudes and pedagogy

Students identified educators’ attitudes and approaches as critical to the success of decolonial education. While students appreciated that many teachers had lived or worked in low-resource contexts, they questioned whether these experiences translated into genuine understanding or merely perpetuated colonial narratives:

We are very lucky to get teachers who … have actually been to and lived in low-income countries … but, at the end of the day, who’s giving the narrative? It’s still from … [their] Western perspectives, so to speak. It would be nice for me … [if] more lectures [had] been given by people who actually still … lived their life [in low-income country contexts]. (Group 3)

Some students also perceived their teachers to overemphasise the challenges faced by LMICs – such as illiteracy, disease or poverty – while overlooking ‘global south success stories’ and local innovations, such as ‘midwifery programmes in the global south’. This was believed to reinforce stereotypical portrayals of LMICs as uniformly struggling or dependent. In response, students proposed incorporating activities such as ‘experience-sharing sessions’ to foster more balanced and nuanced understandings of global health realities.

In addition, some students highlighted instances of insensitive remarks by faculty, which they felt undermined efforts to decolonise education:

It’s surprising that a professor that has worked in Africa … in many places, [can] still not have a very good approach [!] … One of the professors literally said … that the purpose of our [education] program was basically training Black people to go back to Africa to do things better. This colonial notion … [that] white people have the knowledge and they have to teach us [from Africa] how to solve a problem because we are dumb or poor or whatever … this is [a] power relationship. (Group 3)

#### Engaging with Nordic colonial histories and diversifying global health knowledge

Students noted a general lack of engagement with Nordic colonial histories and their influence on contemporary global health education and practice. In some discussions, students perceived the countries in which they studied to have minimal involvement in overseas colonialism. However, they were struck by the persistence of ‘colonial mindsets’ in these contexts, suggesting that such attitudes can endure even in places not widely recognised as former colonial powers.

In other discussions, students mentioned Sámi or Inuit experiences, as well as the Nordic countries’ roles in colonial endeavours, including the transatlantic slave trade. However, they observed a societal tendency to overlook these colonial legacies, while emphasising positive Nordic contributions, such as the advancement of LGBTQ rights.

Students considered engagement with local colonial histories to be a valuable learning opportunity to foster critical reflection among national and international students and to contextualise global health education. Similarly, they advocated for including literature and lectures that explore the history and colonial roots of global health to diversify knowledge systems:

Decolonisation of global health education [means] … bring[ing] in other kinds of knowledge systems … Global health has a history; it goes back to colonialism. It was always taught in a certain way, and that way hasn’t really changed. I mean, there’s been [an] attempt to change, but … bring in knowledge systems from other places in the world, such as Asia, Africa … or we are still being taught in a very Western way. (Group 9)

### Advancing decolonisation through student-led initiatives

#### Reimagining exchange programmes as reciprocal partnerships

Students critiqued the structure and dynamics of their programmes beyond their curricula, focusing on current exchange programmes. They observed that these often reflect and perpetuate colonial power structures, remarking that students from the global south are frequently expected to learn from Nordic countries and apply this knowledge back home, while Nordic students often participate in more observational roles when visiting the global south in an educational capacity:

There are a lot of exchange programmes where students from the global south or underrepresented regions will come to [a Nordic country], where they will learn from the system and professors and other students, and then they’re expected to take that knowledge back home. But … a speaker from [a country in Africa] this year…talked about how students from [a Nordic country] would often come to [the country in Africa] and would often be, sort of, like, tourists in a way. They weren’t necessarily going there in order to learn; they were going … to visit. (Group 4)

They viewed such arrangements as reinforcing superiority and preventing genuinely mutual and transformative learning. Instead, students advocated for equitable, bi-directional collaborations that recognise and value expertise across all contexts.

This call for reciprocal partnerships reflects students’ broader desires for collaborative learning environments grounded in mutual respect and shared knowledge creation. They argued that fostering such exchanges would not only advance decolonisation but also better equip practitioners to address global health disparities more inclusively and effectively.

#### Driving change through student agency and activism

In all discussion groups, students saw themselves as pivotal to driving decolonisation efforts within global health education. They described their role as ‘changemakers’, actively advocating for the inclusion of decolonisation topics in their programmes, where institutional efforts were lacking. Many also mentioned that it is student working groups or organisations that push the decolonisation agenda, with students proactively seeking resources, materials, and experts to fill gaps in their formal education. One participant recounted:

We had this student organisation, and … [in] one of the semesters, we had decolonisation of global health as our focus topic … We found quite different mediums that we could share … so it [decolonisation] is something that we researched on our own time and that we hoped also would someday be a part of the curriculum … We were just so intrigued, and we found a lot of interesting perspectives [on this topic] that we had not seen in our study programme, at least not directly. (Group 4)

In addition to activism, students emphasised the importance of open social discourse and self-reflection as tools for increasing awareness of decolonialisation. Many acknowledged the need to engage in challenging intra- and inter-generational conversations to confront colonial attitudes and to encourage processes of learning and unlearning:

It’s like … a light bulb going on now in your head [afterwards]… Then, in class, when a lecturer projects those ‘Black kids images’ … you can see people, like, I mean, I think the discussions now … are more enlightened. The students themselves are able to see the nuances that some of the lecturers [don’t see], and they’re able to call them out [for colonial attitudes or practices]. And for me … it’s a beautiful thing to see … the growth. (Group 9)

Students articulated a personal commitment to and sense of responsibility for practicing decolonisation in their future roles as global health professionals.

While students were hopeful about the potential for meaningful change within their programmes, they also acknowledged significant barriers. These included limited faculty engagement, inconsistent understanding of decolonisation concepts, and biases in partnerships and collaborations. Despite these challenges, students expressed confidence in their ability to contribute meaningfully to institutional change. Their perspectives highlight both the potential and the limitations of student-led efforts to advance decolonisation within global health education – insights that carry broader relevance for reform efforts in the Nordic region and potentially beyond.

## Discussion

This study explored students’ experiences and perspectives of decolonisation in global health education within the Nordic context, revealing persistent colonial legacies such as power imbalances, eurocentric biases, and institutional barriers. While the scope is deliberately limited to 11 universities in the five Nordic countries, the findings contribute context-specific insights to ongoing global debates on decolonising global health education, specifically by capturing how students perceive and engage with these issues. They illustrate how shared challenges manifest in the Nordic educational context, offering insights into how these dynamics are experienced by students and how their experiences can inform reform efforts both within and beyond the region. These findings echo broader critiques of the discipline and highlight structural and pedagogical challenges that hinder the institutional integration of decolonial perspectives [27,28].

### Reflexivity in interpreting findings

Our interpretation of findings was shaped by the diverse positionalities of the research team, which includes 18 co-authors of different genders, racial, migratory, and ethnic backgrounds, disciplinary expertise, and career stages. All are involved in global health teaching, research, and/or programme coordination at Nordic universities. Several authors helped recruit students from their own institutions and co-moderated FGDs to demonstrate institutional interest and support for student participation. However, the FGDs were primarily facilitated by a PhD candidate – herself a former international master’s student in global health, originally from sub-Saharan Africa, and trained in qualitative methods – whose positionality was intentionally considered to help reduce potential power imbalances and foster openness during data collection. While such imbalances cannot be fully eliminated, her background and facilitation approach likely contributed to a more comfortable environment for participants.

Throughout the analysis and writing process, we engaged in collaborative reflection to critically examine assumptions, ensure that multiple perspectives were considered, and centre student voices in our interpretations. The final presentation of results, and their interpretation, are the product of a collective, iterative process, reflecting the broad positionality of the author team.

### Decolonising global health education: A long-term process

Decolonising global health education is not a quick fix but, much like achieving health equity, a ‘power-saturated long game’ [27]. It requires sustained, critical reflection on entrenched power structures that shape knowledge production, dissemination, and educational practices. Banerjee et al. [29] describe decolonising global health education as a nuanced, ongoing process that involves dismantling colonial structures and addressing power imbalances – far from a token gesture.

Although awareness of the need to decolonise curricula is growing among students and educators in the Nordic context, our findings reveal that these efforts currently lack the institutional commitment and depth required for transformative change. Students highlighted that efforts remain fragmented and often disconnected from the broader institutional culture, which is still shaped by narratives of Nordic neutrality or exceptionalism. Fragmented approaches – such as addressing colonialism and global health history as isolated topics – fail to challenge the deeper structural inequities embedded in the field. A significant challenge lies in confronting aspects of coloniality that are often subtle yet pervasive, such as Eurocentric perspectives entrenched in curricular content, faculty attitudes, and institutional practices. Scholars argue that these practices and mindsets are rooted in ideologies that historically justified enslavement, land dispossession, and cultural erasure in colonised regions. Addressing these issues requires more than reforming curricula; it demands relinquishing the privileges that sustain current hierarchies [17]. This critique is not limited to global health, and similar calls to diversify epistemologies and course materials are emerging across disciplines [30,31]. While this task is daunting, practical steps are possible. Suggestions that have been put forward include increasing representation of faculty with lived experience or professional expertise in the contexts under study, encouraging multilingual engagement in conferences to reduce Anglophone dominance and support diverse forms of participation, expanding definitions of evidence, and exposing students in all settings to diverse learning frameworks [17].

Use of terminology also plays a pivotal role in reinforcing power imbalances in global health. Terms like ‘HICs versus LMICs’ or ‘resource-rich versus resource-limited’ perpetuate binary hierarchies, portraying the ‘global north’ as superior while framing the ‘global south’ primarily in terms of deficits [32]. These classifications obscure the diversity, agency, and innovations of countries and communities grouped under broad labels like ‘the global south.’ Questioning and critically examining such language is essential in global health education because it shapes how challenges are framed and how solutions are conceptualised.

Students also critiqued the structure and assumptions underpinning international learning arrangements within their programmes. They observed that educational opportunities served different functions depending on students’ regional backgrounds. Those from the global south were often positioned as recipients of knowledge and skills to be applied back home, while students from the Nordic region or other high-income settings typically engaged with LMIC contexts in observational or short-term project roles. These asymmetrical dynamics were seen to reinforce existing power imbalances rather than fostering genuine mutual learning. Students called for the redesign of such initiatives to promote more reciprocal and collaborative learning experiences, where all participants are recognised as both teachers and learners. Building equitable partnerships has been identified as essential for advancing transformative global health education [33].

Decolonising Nordic global health education, as reflected in the perspectives of the students in this study, will require more than surface-level reforms. While our findings are limited to student voices, they point to the need for sustained reflection, systemic change, and a commitment to dismantling the ideological and structural legacies of colonialism that continue to shape the field [34].

### Missed opportunities: addressing Nordic colonial histories in education

Our findings reveal an overlooked opportunity to integrate Nordic colonial histories into global health education. Students reported limited exposure to critical discussions about the Nordic states’ relationships with Indigenous peoples, such as the Sámi and Inuit, as well as their involvement in overseas colonial activities, including Denmark’s expansion in the Caribbean and Africa. This aligns with broader critiques of Nordic education, where coloniality is often downplayed or ignored, reinforcing notions of Nordic exceptionalism and white innocence, and where emotional resistance to engaging with race and historical complicity persists [20,35,36]. This omission is particularly relevant for Nordic global health programmes, where many international students arrive with expectations of progressive, equity-focused education, but instead encounter limited engagement with colonial legacies in the classroom. Since north-south power imbalances often evoke ‘emotionally charged’ discussions shaped by participants’ diverse backgrounds, reflecting on coloniality within the Nordic region itself could provide a shared starting point for critical reflection on global health power dynamics.

Encouraging discussion about whose knowledge is valued and taught, and whose priorities shape health system design, could prompt Nordic global health programmes to engage more critically with local inequities while simultaneously addressing global power structures. By fostering this dual perspective, these programmes could better equip students to analyse and challenge the hierarchies underpinning both regional and global health inequities.

### Balancing global health priorities: Nordic programmes and resource allocation

The Nordic region hosts numerous global health programmes, including at the 11 universities involved in this study. This concentration of global health educational resources raises important questions about where the priorities lie in addressing global health inequities. Students in our study debated whether such programmes genuinely align with principles of equity, or whether they risk reinforcing knowledge hierarchies and imbalances in global health leadership and educational opportunities between HICs and LMICs. Scholars advocating for a decolonial shift have called for increased investment in LMIC-based institutions to expand access to global health education through locally led programmes. This approach recognises the existing strengths and innovations within many LMIC institutions, while emphasising the need for more equitable distribution of educational resources and leadership opportunities in the field [8,17]. At the same time, others argue that strong programmes in HIC settings, such as those in the Nordic region, remain important for addressing both global and local inequities – for example, by fostering inclusive learning environments and cross-contextual understanding.

The growing global demand for global health training underscores the need to expand educational capacity in diverse settings. Achieving this will require sustained investment and long-term partnerships built on mutual trust and respect, with shared commitments to equity and locally relevant capacity-strengthening [11,17]. Expanding opportunities for local education and leadership may also help address the critical shortage of highly trained professionals needed to respond to disproportionately high disease burdens in many LMICs. Yet progress remains uneven, constrained by structural barriers in funding, accreditation, and international collaboration.

Rather than treating the expansion of LMIC-based programmes and the continuation of HIC-based programmes as mutually exclusive priorities, Nordic institutions can enhance their contributions to global health by aligning more intentionally with equity and sustainability goals. As students in this study suggested, this includes fostering more equitable collaborations with LMIC partners and addressing enduring inequities within their own national contexts, particularly those affecting indigenous populations. Reframing global health as a shared and universal concern, encompassing challenges across all regions, offers a pathway toward more inclusive, context-responsive, and transformative educational approaches.

### A framework for student agency and transformative change

Despite institutional barriers, students in this study emerged as key agents of change, advocating for the inclusion of decolonial perspectives within their programmes. However, many expressed feelings of isolation in these efforts. This mirrors broader dynamics in which structurally weaker actors leverage alternative forms of power – such as network power and discursive strategies – to challenge dominant narratives [27]. To address this, universities must actively collaborate with students to co-create curricula that incorporate diverse perspectives and support student-led initiatives, such as seminars, campaigns, or social media projects. Embedding reflexivity throughout the curriculum is another critical step, drawing on pedagogical approaches that encourage both students and educators to critically examine their own positionality within the historical legacies that shape global health.

As Garba et al. [37] highlight, creating spaces for students to reflect on their motivations for engaging in global health is essential. This could involve mandatory courses on the historical foundations of global health and a critical interrogation of the language and terms used in global health discourse. Framing decolonisation as a ‘threshold concept’ (33) further emphasises its potential to initiate deep, potentially transformative learning that challenges conventional understandings and supports students’ critical engagement with global power dynamics.

### Recommendations for decolonising Nordic global health education

Based on students’ perspectives in this study, we propose recommendations for Nordic and other educational institutions to consider (Table 4). These offer practical steps for integrating decolonial perspectives into global health teaching, research, and institutional practices.

**Table 4.** Recommendations for educational institutions to decolonise their global health programmes.

Focus areas	Suggestions for integration into education
Framing, terminology and definitions	Critically assess and revise the terminology used in global health to avoid reinforcing biases.
History of global health	Include mandatory courses on the historical development of global health and its colonial legacy.
Local colonial histories	Engage students in discussions about local colonial histories, including Nordic ones, to draw meaningful parallels with global dynamics.
Impact of coloniality on global health practices	Offer workshops to explore how colonial-era power structures continue to shape global health research, partnerships, and policies.
Diversifying educational content	Incorporate literature, multimedia (e.g. documentaries, podcasts), and case studies from diverse geographic and cultural contexts to reduce Eurocentrism and expand epistemic diversity, offering students a deeper and more inclusive understanding of global health.
Involvement of people in low- and middle-income countries and underserved populations in teaching and supervision	Incorporate a wider spectrum research colleagues in teaching and supervision through video recordings, online collaborations or guest lectures.
Integrate reflexivity in global health	Embed continuous reflexivity exercises into curricula to encourage students and faculty to critically examine positionality and privilege.
Reciprocal exchange programmes	Develop equitable, two-way exchange programmes that foster mutual learning and respect; ensure opportunities for bidirectional exposure and learning.
Faculty training	Provide faculty training on decoloniality and inclusive teaching practices to enhance their capacity to address these topics.
Student-led initiatives	Support and amplify student-led decolonisation activities, such as seminars, blogs or workshops, to encourage their advocacy efforts.
Student involvement in curriculum development	Involve students in co-creating curricula to ensure diverse perspectives with greater relevance to global health education.

#### Study strengths and limitations

This study’s key strength lies in its multi-institutional approach, capturing diverse perspectives from students across 11 universities in five Nordic countries. The use of FGDs facilitated rich discussions, revealing complex and, at times, diverging views on decolonisation in global health education. To support trustworthiness, we used multiple coders, an iterative coding process, and extensive team discussions to ensure analytic consistency. The involvement of a diverse group of co-authors in the analysis and interpretation contributed to a transparent and reflexive process, helping us to remain attentive to power dynamics and positionality while centring students’ voices in our interpretations.

Preliminary findings were presented and discussed at a network meeting involving institutional colleagues who were not formally involved in the study as well as some of the FGD co-moderators. While these discussions enriched our reflexive engagement with the material, they were not part of a formal validation process and should be interpreted accordingly.

However, several limitations should be noted. Reliance on self-selected participants may have introduced bias, as those with strong views on decolonisation might have been more likely to participate. Limited representation from regions such as Latin America, the Caribbean, and Eastern Europe may also have further restricted the diversity of perspectives captured. Moreover, although the project team included programme directors and faculty from the participating programmes, we did not undertake a systematic curriculum review or interview educators, limiting our ability to formally triangulate student perspectives with institutional or faculty viewpoints. Future research could explore educators’ perspectives and examine the impact of recent or upcoming decolonisation initiatives, offering a more comprehensive understanding of the evolving landscape of global health education in the Nordic context.

## Conclusions

This study underscores that decolonising global health education is a complex and ongoing process, requiring sustained institutional commitment, critical reflection, and structural change. The structural barriers identified by students – including persistent power asymmetries in global health policy, funding, and governance – reflect the broader systemic challenges that must be addressed to advance equity and inclusion in the field.

Based on students’ experiences and perceptions of how decolonisation is expressed within their programmes, we propose strategies to guide the development of more inclusive, reflexive, and contextually relevant approaches to global health education in the Nordic region. Although our findings are limited to student perspectives from the Nordic context, they may inform reflection, dialogue, and reform efforts in global health education elsewhere. Engaging with these insights offers an opportunity to foster more equitable and transformative educational practices across diverse settings.

## Data Availability

This study adheres to Tampere University’s data policy, which promotes free access to publicly funded research data while ensuring confidentiality. In alignment with the Finnish Social Science Data Archive (FSD) and the FAIR principles (Findable, Accessible, Interoperable, and Reusable), anonymised data will be made openly available through the FSD whenever possible, in accordance with participants’ preferences and ethical considerations. Due to confidentiality constraints, raw data containing identifying information will not be shared.
